# Supplementary material for: Dynalign II: common secondary structure prediction for RNA homologs with domain insertions
Source: Nucleic Acids Res. 2014 Nov 21;42(22):13939–48. doi: 10.1093/nar/gku1172 (PMC4267632; doi:10.1093/nar/gku1172)
Supplement: SUPPLEMENTARY DATA [file supp_gku1172_nar-02021-z-2014-File012.zip › manual/javadoc/RNAstructure_java_interface/source/menus/RolloverMenu.RolloverListener.html]

RolloverMenu.RolloverListener


JavaScript is disabled on your browser.


- Overview
- Package
- Class
- Tree
- Index
- Help

- Prev Class
- Next Class

- Frames
- No Frames

- All Classes

- Summary:
- Nested |
- Field |
- Constr |
- Method

- Detail:
- Field |
- Constr |
- Method


RNAstructure\_java\_interface.source.menus

## Class RolloverMenu.RolloverListener

- java.lang.Object
- - RNAstructure\_java\_interface.source.menus.RolloverMenu.RolloverListener

- All Implemented Interfaces:
  :   MouseListener, Serializable, EventListener

  Enclosing class:
  :   RolloverMenu

  ---

    

  ```
  public class RolloverMenu.RolloverListener
  extends Object
  implements MouseListener, Serializable
  ```

  An inner class that creates a rollover listener for menus in the
  RNAstructure GUI.
    
    
  The rollover text is placed in the message bar at the bottom of the main
  application frame, whenever a user interacts with the menu item this
  listener is attached to, and removed to make way for the default message
  text when the user stops interacting with the menu item.

  See Also:
  :   Serialized Form

- - ### Field Summary

    Fields

    | Modifier and Type | Field and Description |
    | --- | --- |
    | `private String` | `rollover` The rollover text for this listener. |
    | `private static long` | `serialVersionUID` |
  - ### Constructor Summary

    Constructors

    Constructor and Description

    `RolloverMenu.RolloverListener(String text)`

    Constructor.
  - ### Method Summary

    Methods

    | Modifier and Type | Method and Description |
    | --- | --- |
    | `void` | `mouseClicked(MouseEvent e)` |
    | `void` | `mouseEntered(MouseEvent e)` |
    | `void` | `mouseExited(MouseEvent e)` |
    | `void` | `mousePressed(MouseEvent e)` |
    | `void` | `mouseReleased(MouseEvent e)` |
    | `private void` | `reset()` Reset the main frame message bar text to its default. |
    | `private void` | `set()` Set the main frame message bar text to this listener's specific rollover text. |

    - ### Methods inherited from class java.lang.Object

      `clone, equals, finalize, getClass, hashCode, notify, notifyAll, toString, wait, wait, wait`

- - ### Field Detail


    - #### rollover

      ```
      private String rollover
      ```

      The rollover text for this listener.


    - #### serialVersionUID

      ```
      private static final long serialVersionUID
      ```

      See Also:
      :   Constant Field Values
  - ### Constructor Detail


    - #### RolloverMenu.RolloverListener

      ```
      public RolloverMenu.RolloverListener(String text)
      ```

      Constructor.

      Parameters:
      :   `text` - The rollover text.
  - ### Method Detail


    - #### mouseClicked

      ```
      public void mouseClicked(MouseEvent e)
      ```

      **Specified by:**
      :   `mouseClicked` in interface `MouseListener`


    - #### mouseEntered

      ```
      public void mouseEntered(MouseEvent e)
      ```

      **Specified by:**
      :   `mouseEntered` in interface `MouseListener`


    - #### mouseExited

      ```
      public void mouseExited(MouseEvent e)
      ```

      **Specified by:**
      :   `mouseExited` in interface `MouseListener`


    - #### mousePressed

      ```
      public void mousePressed(MouseEvent e)
      ```

      **Specified by:**
      :   `mousePressed` in interface `MouseListener`


    - #### mouseReleased

      ```
      public void mouseReleased(MouseEvent e)
      ```

      **Specified by:**
      :   `mouseReleased` in interface `MouseListener`


    - #### reset

      ```
      private void reset()
      ```

      Reset the main frame message bar text to its default.


    - #### set

      ```
      private void set()
      ```

      Set the main frame message bar text to this listener's specific
      rollover text.


- Overview
- Package
- Class
- Tree
- Index
- Help

- Prev Class
- Next Class

- Frames
- No Frames

- All Classes

- Summary:
- Nested |
- Field |
- Constr |
- Method

- Detail:
- Field |
- Constr |
- Method
